# Grade progression in urothelial carcinoma can occur with high or low mutational homology: a first-step toward tumor-specific care in initial low-grade bladder cancer

**DOI:** 10.18632/oncotarget.24072

**Published:** 2018-01-06

**Authors:** Ralf Kittler, Christine Shiang, Ryan Hutchinson, Rahul K. Kollipara, Payal Kapur, Francis Franto, Yair Lotan

**Affiliations:** ^1^ Eugene McDermott Center for Human Growth and Development, University of Texas Southwestern Medical Center, Dallas, Texas, USA; ^2^ Department of Urology, University of Texas Southwestern Medical Center, Dallas, Texas, USA; ^3^ Department of Pathology, University of Texas Southwestern Medical Center, Dallas, Texas, USA

**Keywords:** bladder cancer genomics, low grade, progression

## Abstract

**Purpose:**

Low-grade (LG) urothelial carcinomas of the bladder (UCB) are common malignancies that are costly to surveil and rarely progress to life threatening, high-grade (HG) malignancies. It is unknown if the progression of LG to HG is a result of second primary tumors or transformation of existing LG tumors. We examined tumor genetics in patients with grade progression in urothelial carcinoma and compared to patients with no progression.

**Results:**

Five patients were identified with progression. Median time from initial LG diagnosis to HG diagnosis in those experiencing progression was 19 months. Progression with both high and low mutational homology was identified. Gene alterations associated with tumor grade progression in initial low grade tumors include FBN3, CIT and HECTD4.

**Materials and Methods:**

An institutional cancer database at a tertiary referral center in the United States identified patients who progressed from LG to HG UCB. Histologic re-review was performed by a genitourinary pathologist. Whole exome sequencing with correction for germline mutations by buffy coat subtraction was performed. Mutations were assessed between LG tumors and subsequent same-patient HG tumors and for LG patients who did not progress. Individual genes were assessed as potential predictors of risk for progression.

**Conclusions:**

Tumor grade progression occurred with both high mutational homology and low mutational homology, which may represent both true tumor progression and de-novo growth. Validation of the identified tumor genes that appeared associated with progression may provide a clinically valuable tool to providers managing patients with LG urothelial carcinomas.

## INTRODUCTION

Bladder cancer is the fifth most common malignancy in the United States [1]. In 2016, more than 76,000 new cases of bladder neoplasms were diagnosed in the United States [1]. Seventy-five percent of new tumors are non-muscle invasive bladder cancers (NMIBC) most of which are urothelial cell carcinomas [2]. Seventy percent of non-muscle-invasive bladder cancers are Ta and non-invasive, low-grade tumors have a high 5-year recurrence rate (31 to 78%), low risk of progression to high-grade disease, and require cystoscopy for detection of tumor recurrence [3–6]. Grade progression specifically is a critical transition point in urothelial carcinomas, with true low grade malignancies having a very low metastatic potential while high grade tumors, even those progressing from low grade, carry significant risk of morbidity and mortality [7]. This paradox of high recurrence and low risk of disease progression in initial low grade bladder tumors contributes to surveillance regimens that appear ineffective at identifying at-risk patients and at care costs that are among the highest on a per-patient basis [8]. Previous studies have examined urothelial carcinoma genetics in recurrence using earlier techniques or with an emphasis on multiple synchronous tumors [9–11].

Improvements in future management depend on identifying factors that can triage patients that require intensive follow-up versus those that are at low risk of disease progression. One of the challenges is predicting which patients will develop high grade disease since low grade non-invasive tumors are not lethal but high grade tumors can metastasize leading to death. To date, there is not a validated genomic predictor panel for patients at risk of low-to high-grade disease progression. A key question that arises is whether disease progression is a consequence of true progression from low-to high-grade disease or if a second primary event occurs due to changes in the urothelium from carcinogen exposure. We sought to study this question through whole exome interrogation of patients with low-grade tumors that subsequently developed high grade disease to identify potential predictors of disease progression. We also compared mutations in these tumors to low grade tumors in patients that did not progress.

## RESULTS

**Figure 1:**
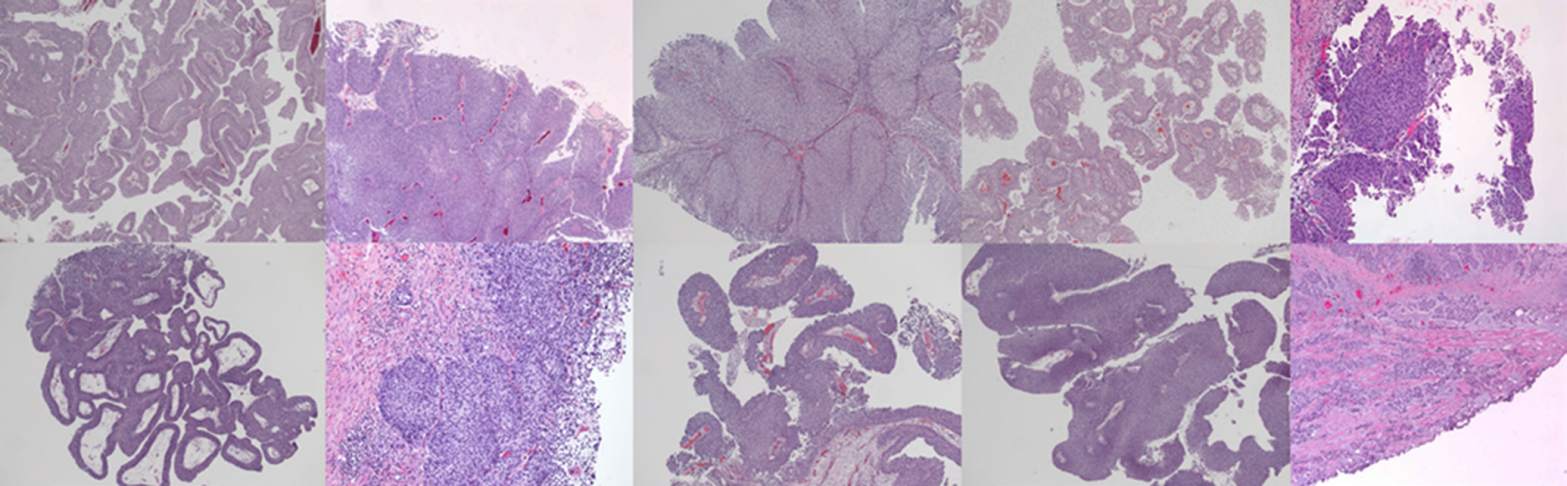
Representative micrographs of patients with progression (top low grade initial diagnoses, bottom high grade recurrences)

We sequenced the exomes of 27 samples that comprised matched germline (buffy coat), low grade tumor and high grade tumor samples with median followup of 19 months for progressors and median non-progressor followup of 51 months. These samples were as follows: five patients (PT1-5) with grade progression (five low grade and five high grade tumors sequenced) and six patients with (PT6-11) recurrent low grade tumors (initial low grade tumors sequenced). The median age at initial diagnosis for patients with progression was 68 (range 34 to 86) and without progression was 57 (range 55 to 65). Gender distribution was 73% (8 of 11) male. Of the patients who progressed from low grade Ta, the stages and grades of tumors they progressed to was as follows: high-grade Ta (*n* = 2), carcinoma-in-situ (*n* = 1), high-grade T1 (*n* = 1) and high-grade T2 (*n* = 1). For patients without grade progression the median number of recurrences was 2 (range 1 to 5) with a median followup of 51 months (range 17 to 56). Additional clinicopathologic data including grade, stage, risk factors and tumor location at surgery are contained in Supplementary Table 1. Representative histologies for initial Ta low grade and subsequent high grade lesions in these patients are presented as Figure 1. We found the exome data of excellent quality in terms of coverage (e.g. average of at least 20X coverage of the targeted bases is 96.9%) for all samples (see Supplementary Table 2), which is in particular critical for DNA obtained from FFPE samples.

**Figure 2:**
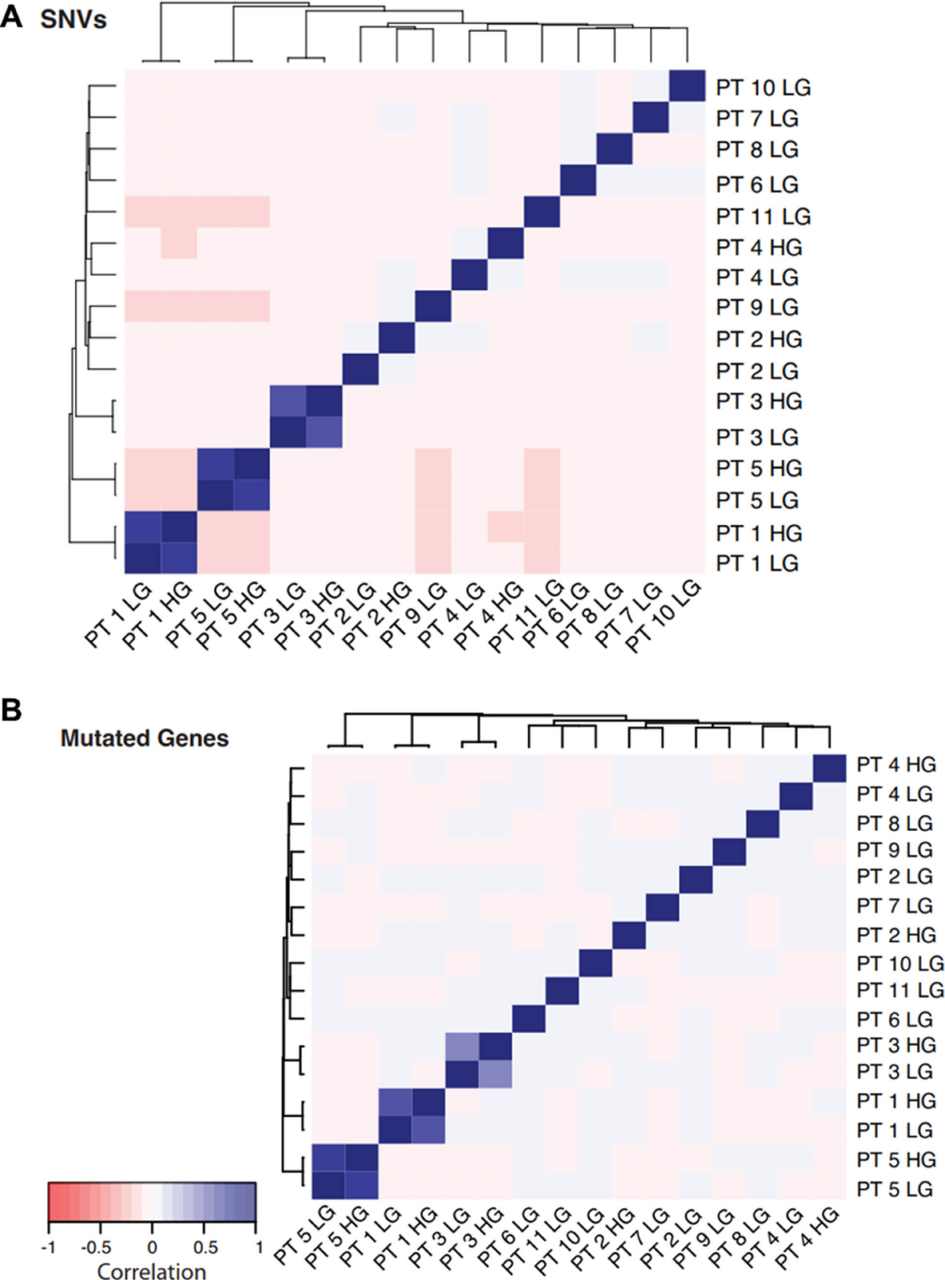
Heat maps of overlapping mutations found in cohort

**Figure 3:**
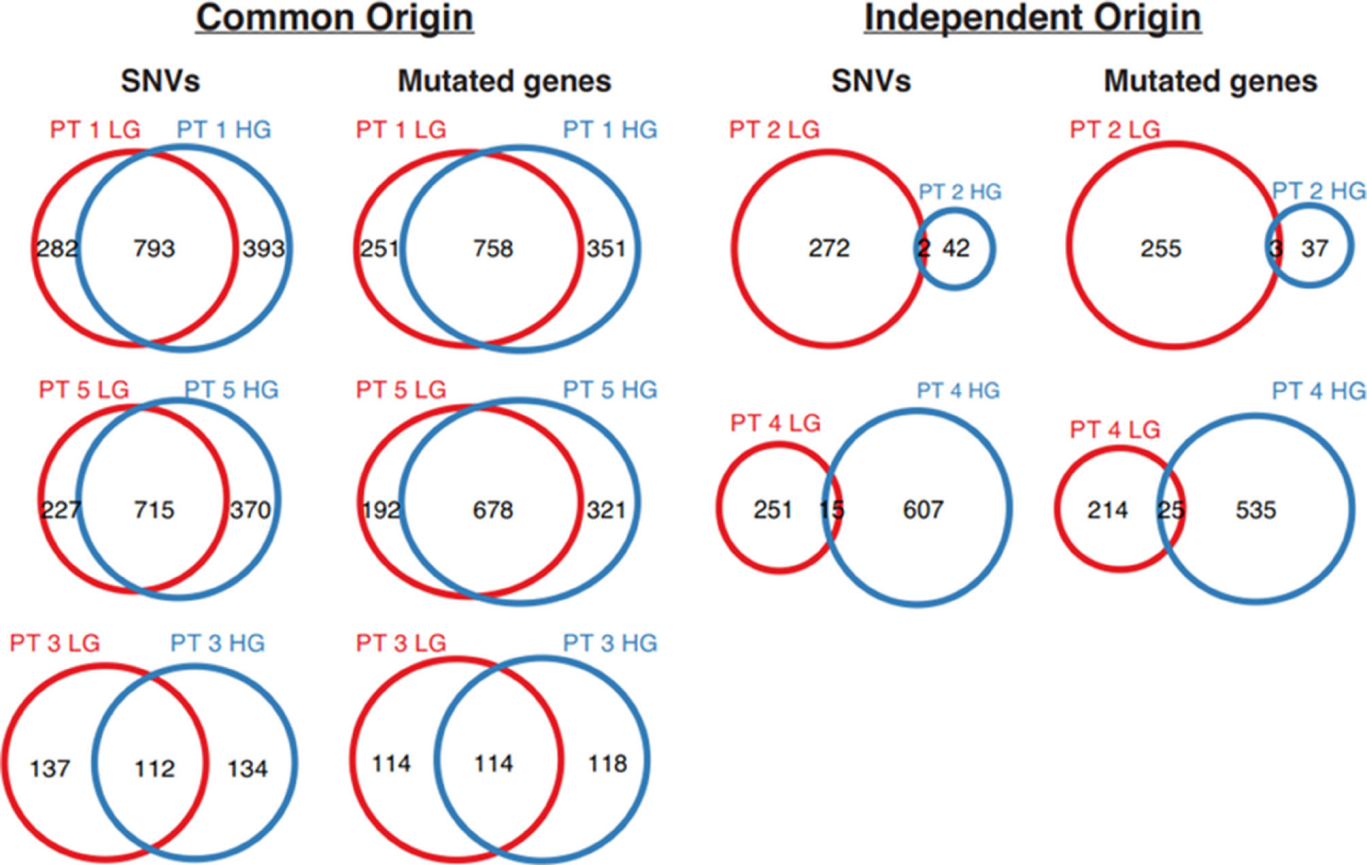
Venn diagrams of overlapping mutations in patients with grade progression

The unsupervised clustering of genetic similarity based on shared mutated genes or single nucleotide variants (SNVs, i.e. somatic point mutations) of the tumors revealed that for three of the tumor pairs the low grade and high grade had a high degree of genetic similarity (Figure 2). While the small sample numbers limit the power of statistical analysis, we found a similar number of somatic mutations in the low grade and high grade of these matched pairs with proposed common origin of which at least ~50% were shared, while the two sample pairs with proposed independent origin differed markedly in the number of somatic mutations (Figure 3). One subject (patient #4) displayed some characteristics of both independent and common origin with 15 SNVs common to both tumors. We further investigated this by examining the common genes affected by these SNVs (Supplementary Table 3). To our knowledge one of these genes, STAG2, would be relevant to oncogenesis within urothelial carcinoma but has not been described as a driver mutation. The final stage to which these tumors progressed did not appear to correlate with independent or common origin; common origin patients progressed to high-grade Ta (*n* = 2) and high-grade T1 (n = 1) tumors and independent origin patients progressed to CIS (*n* = 1) and T2 (*n* = 1) tumors.

**Figure 4:**
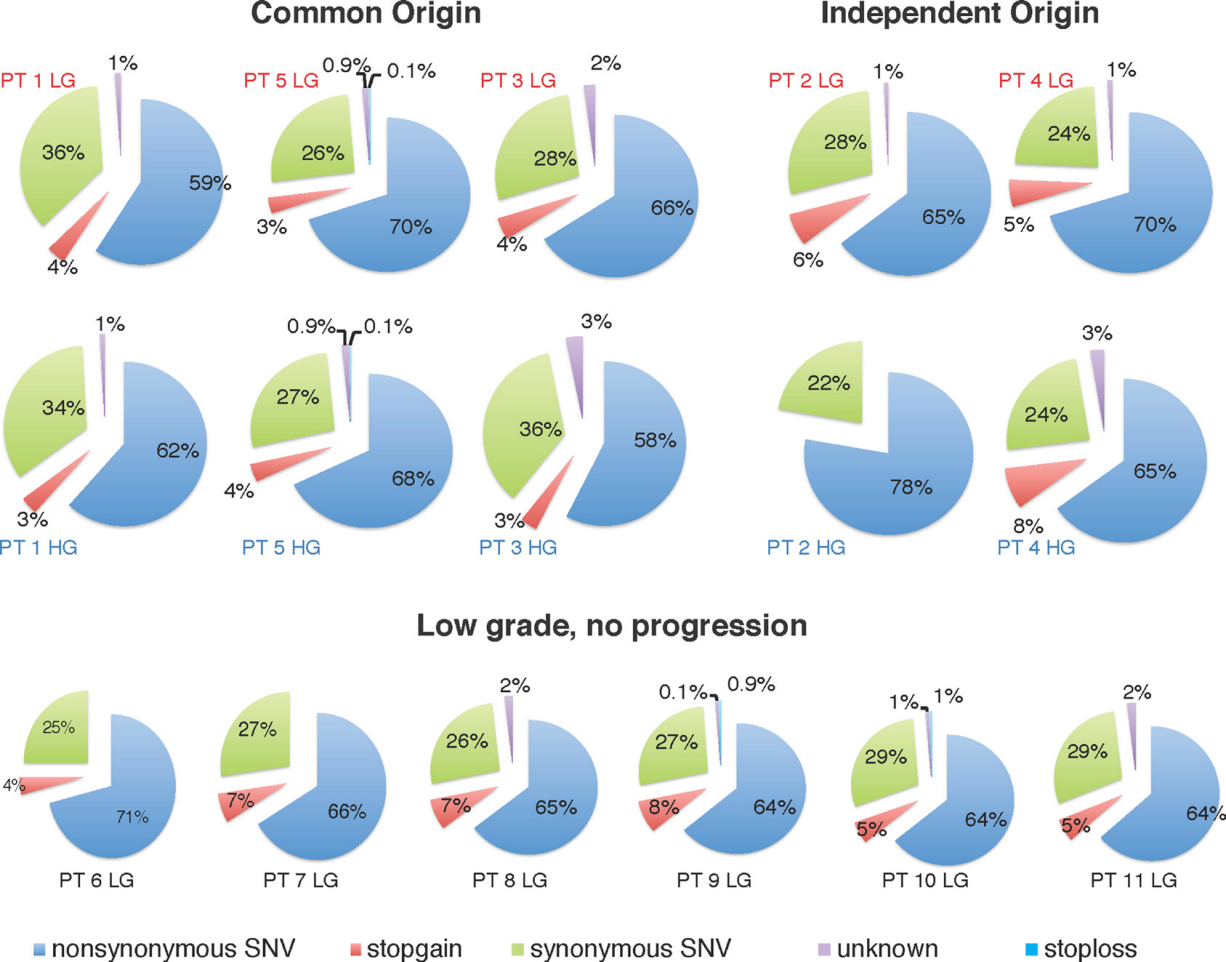
Mutation types for the patient cohort

When we analyzed the impact of the point mutations on gene products we found that most SNVs were non-synonymous (Figure 4), which is commonly observed for most solid tumors where passenger mutations are prevalent.

**Figure 5:**
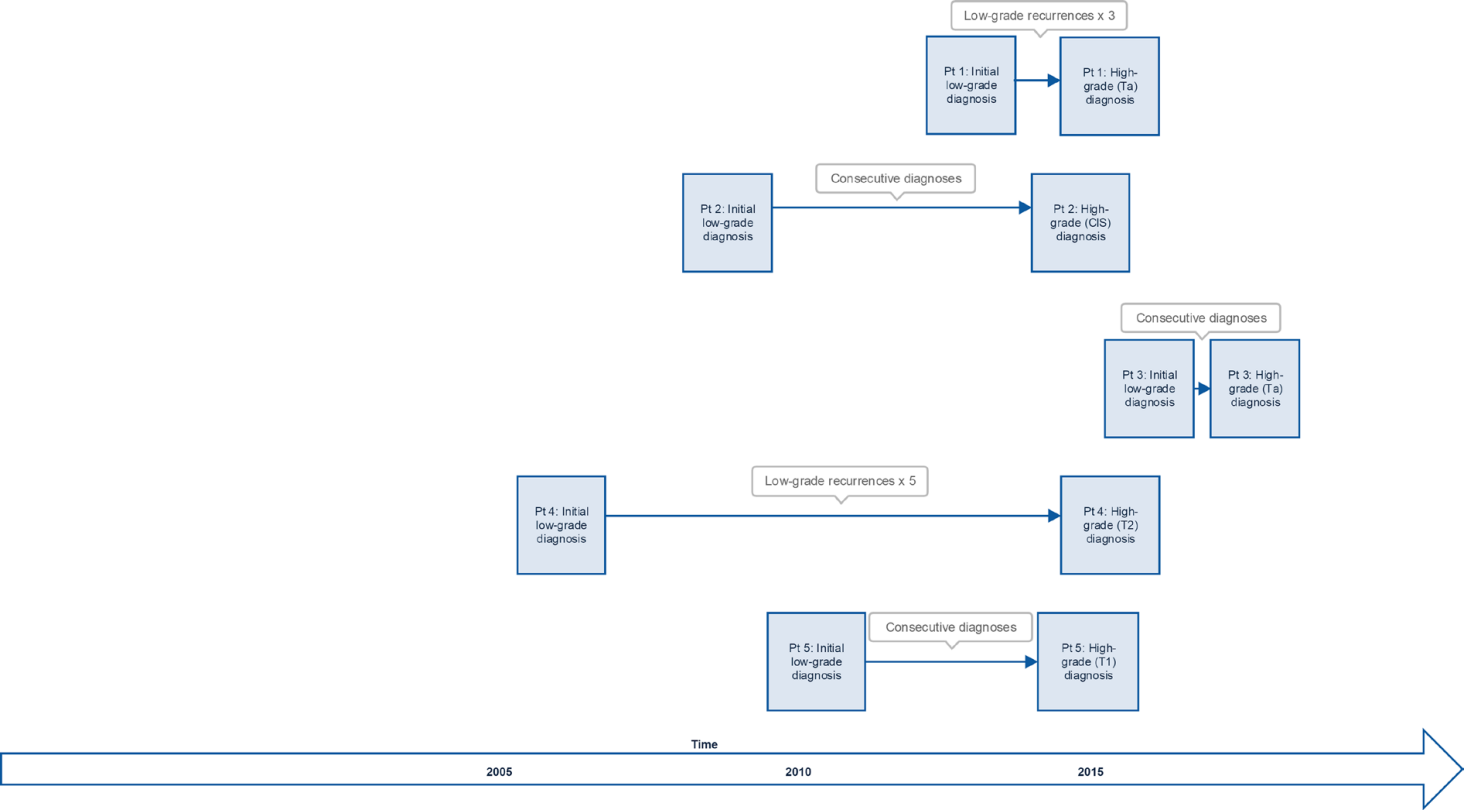
Time course for patients with progression

In patients who progressed from low grade to high grade tumors, twenty mutually exclusive (present in most low grade that progressed but not present in any recurrent low grade who did not progress) gene alterations were noted. A visual representation of temporal progression is presented as Figure 5. Conversely, fewer gene alterations were mutually exclusive to recurrent low grade tumors (found in patients with low grade tumors which recurred as low grade but not found in low grade tumors that progressed). These are summarized in Table 1. Multiple mutually exclusive genes were found mutated only in progressors and, conversely, only in non-progressors. The largest differences were found in the following genes, with their number of mutations in progressors and non-progressors listed in parentheses: FBN3 (5,0), CIT (4,0), HECTD4 (4,0), COL22A1 (0,3), ATP13A5 (0,3) and FAM186A (0,3). Specific breakdown of gene function class is presented in Table 2. The most frequently noted classes mutated in subsequently-progressive low grade tumors but not in non-progressive tumors were mutations in cytoskeletal and cell cycle regulation genes.

**Table 1:** Mutually exclusive gene mutations for progressors and non-progressors

Mutually exclusive genes occurring in majority (3 or greater) samples assessed			
Gene	Number of occurrences in Low Grade tumors with grade progression	Number of occurrences in Low Grade tumors without grade progression	Chromosomal Location of Gene
FBN3	5	0	19p13.2
CIT	4	0	12q24.23
HECTD4	4	0	12q24.13
ATR	3	0	3q23
BAHCC1	3	0	17q25.3
DOCK2	3	0	5q35.1
MICAL3	3	0	22q11.21
TECPR2	3	0	14q34.31
WDR4	3	0	21q22.3
C1orf112	3	0	1q24.2
CTNNA1	3	0	5q31.2
ENGASE	3	0	17q25.3
GTF3C1	3	0	16p12.1
HSPG2	3	0	1p36.12
ICAM5	3	0	19p13.2
MAP4	3	0	3p21.31
MOCOS	3	0	18q12.2
POSTN	3	0	13q13.3
HDAC9	3	0	7p21.1
UTRN	3	0	6q24.2
COL22A1	0	3	8q24.23
ATP13A5	0	3	3q29
FAM186A	0	3	12q13.12

**Table 2:** Types of genes mutated in initial low grade urothelial tumors that progressed

Mutations Exclusive to Tumors with Grade Progression		
Connective Tissue and Cytoskeleton	FBN3	
	BAHCC1	
	DOCK2	
	CTNNA1	
	HSPG2	
	ICAM5	
	POSTN	
	UTRN	
**Cell Cycle and Transcription Regulation**	CIT	
	ATR	
	MICAL3	
	WDR4	
	GTF3C1	
	MAP4	
	HDAC9	
**Transmembrane and Signaling**	HECTD4	
	MOCOS	
**Autophagy and Lysis**	TECPR2	
	ENGASE	
**Uncharacterized**	C1orf112	

## DISCUSSION

**Figure 6:**
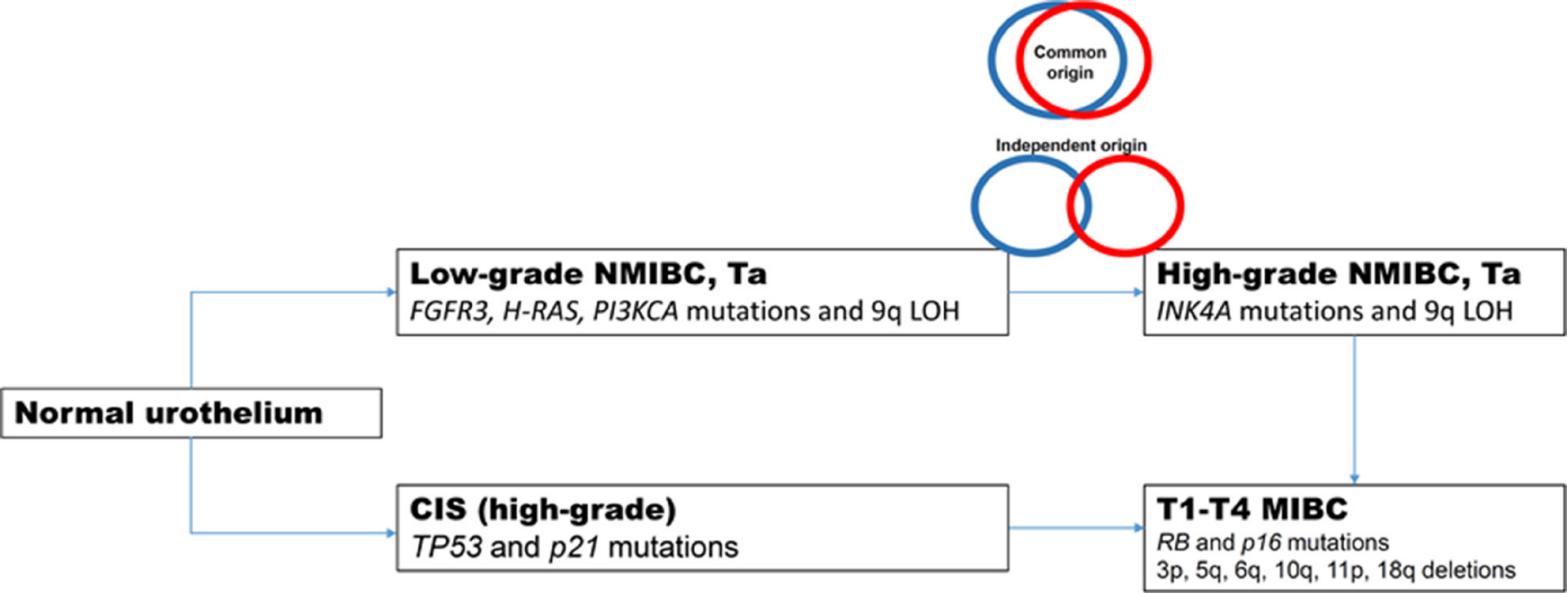
A potential genetic pathway for the generation of high-grade, invasive urothelial carcinomas

Non-muscle invasive bladder cancer (NMIBC) is subdivided into 2 unique groups on the basis of observed phenotypic appearance. The first group, low grade noninvasive (Ta) UCB have a typical papillary appearance and recur frequently with a small percentage progressing to a higher stage. These tumors are characterized by oncogenic mutations in *FGFR3, H-RAS,* and *PI3KCA* [2, 12]. High-grade papillary lesions are typified by deletions in chromosome 9q and mutations in *INK4A* and have an increased rate of progression to invasive disease in comparison to low-grade disease [2]. The second group consists of carcinoma *in situ* (CIS), a high-grade lesion characterized by *TP53* and *p21* mutations with a 60-80% progression rate to invasive disease involving *RB* and *p16* (T1-T4) [2]. A visualization of this is shown in Figure 6.

For bladder cancer, grade is more important than stage in predicting tumor progression. Specific molecular events may be responsible for the transition between low- and high-grade disease within the spectrum of noninvasive papillary urothelial carcinomas. Despite the identification of these molecular alterations inherent to each subtype of bladder cancer, there is a lack of understanding of the molecular events that underlie the transition from low- to high-grade disease. It is possible that low grade tumors develop new mutations that result in a transition to HG disease. Prior studies have examined phylogenetic trees of disease evolution in urothelial carcinoma and found longer ancestral branches in non-progressive disease, consistent with a higher proportion of field mutations in these tumors [13]. However, there is also a possibility that second primary tumors develop independent of the original low grade tumor as a consequence of the underlying exposure of the entire urothelium to carcinogens.

Using paired, same-patient samples, we identified that both of these hypotheses occur with transition of some LG tumors to high grade disease with a high concordance of mutations. Similarly, some patients exhibited mostly unique mutations with little similarity between the LG and HG tumors. Due to the small number of tumors it is not possible to determine if there is a common mutation that can predict the likelihood of a progression event which is rare for LG tumors occurring less than 5% of the time.

However, in patients who progressed from low grade to high grade tumors, twenty mutually exclusive (present in most low grade that progressed but not present in any recurrent low grade who did not progress) gene alterations were noted. Future directions may include interrogating larger numbers of paired sample and further validation of overlapping sets of mutations.

Percentage of targeted bases at 20X coverage is a commonly used quality measure for exome sequencing with values > 90% considered excellent. The mean coverage of our exome sequence data was ~130X and our percentage of 50X coverage was 85.6%, which conforms to the requirements of deep exome coverage. We are confident that our whole exome data has sufficient coverage for the mutation analysis performed in this study.

Genomic-based signatures aimed at identifying bladder cancer patients at risk for recurrence have been evaluated since 2006. Early efforts used an oligonucleotide array on 105 bladder tumors and predicted overall survival in all bladder cancer patients in the study and those with muscle-invasive disease [14]. A 4-marker panel was shown to detect differences in recurrence and survival across all stages of urothelial bladder cancer, with a similar approach used in a 24-gene panel and multivariable analysis which identified predictors of recurrence and progression in patients presenting initially with high-grade Ta tumors [15, 16]. Other studies used high-throughput profiling strategies to detect gene signatures of disease progression from NMIBC to muscle-invasive disease [17]. More recently, GenomeDx Biosciences has developed a genomic classifier, KNN51, to predict lymph node metastasis during radical cystectomy in patients with muscle-invasive bladder cancer. Early studies indicate that the KNN51 genomic classifier outperforms clinical classifiers [18]. In NMIBC, the combination of common SNPs and clinicopathologic parameters slightly improved prediction of time-to-first-recurrence, but not time-to-progression [19]. These studies indicate that investigations of muscle invasive disease are well-underway and more studies are needed to understand NMIBC especially LG disease which while rarely fatal is common. Recently, sequencing techniques have evolved to allow greater interrogation of cellular genetic and transcription status, even in tissue that is archival. In 2014 Liu et al published their results using RNAseq on archival urothelial tumors fixed in paraffin and were able to demonstrate a high correlation with fresh tissue and differential homogeneity between high and low grade tumors [20]. Similar techniques were used in recently presented work on non-muscle invasive bladder cancer which compared genomic characterization of these tumors to histologic assessment with respect to reproducibility and ultimate clinical outcomes. The authors identified three distinct molecular classes of NMIBC which were more readily reproducible than standard pathologic analysis and tracked well with actual tumor behavior [21].

Low-grade, stage Ta tumors are rarely life-threatening. 70% of patients experience recurrent low-grade tumors, with low rates of progression, 5–15%, to a higher grade or stage [2, 22]. Hernandez et al reported a median follow-up of 6 years for patients undergoing active surveillance, with 79.3% patients that had not progressed in grade and 86.4% had not progressed in stage [4]. However, routine surveillance with cystoscopy remains the mainstay for detection of the minority of patients that may develop recurrence or progression. The high cost of surveillance makes bladder cancer the most costly cancer among the elderly, with approximately $4 billion in treatment costs [23, 24].

Previous studies have found cost-savings when alternating the use of urine tumor markers with cystoscopy and cytology [25]. In comparison, the addition of urine tumor markers, FISH, and cytology to cystoscopy increased costs without improving the sensitivity of detecting invasive disease [8]. The use of predictive markers in clinical decisions for urothelial bladder cancer has been slow due to inadequate independent validation, lack of reference standards, and limited prospective randomized trials. In this study, we report on the major mutational events responsible for the highly recurrent low-grade tumors of NMIBC and contribute to the field of genomic tools that can be used in future validation.

This study provides early information about gene mutations from a small cohort and therefore may be subject to several limitations. We were unable to examine time-based changes in mutational burden except at the two discreet time points examined. This lack of granularity cannot rule out distant common origin from a field defect for tumors believed to be of independent origin, especially mutations that may be germaine to etiologic agents like smoking. We recognize the small number of patients evaluated which is in large part due to the uncommon nature of progression in low grade disease. However, since this is the most important risk for lethality in a low grade patient it is important to evaluate this question. Even with small numbers of patients, we found distinct differences between those patients with rare common mutations and those with very high proportion of common mutations. A larger sample size would help elucidate the frequency of having true tumor progression versus de-novo growth but will not negate the finding of both possibilities. In using whole exome analysis on a discovery cohort, there is an intrinsic risk of overfitting. Further multi-institutional validation studies will be needed to determine which genes identified, if any, represent an optimal predictor panel. Further avenues for study could also include serial sequencing of multiply recurrent low grade tumors with or without progression to further elucidate a potentially targetable turning point in the progression of urothelial carcinomas. Differential times-to-event for the patients with progression events could also confound the identification of patients with de novo tumors versus true tumor progression.

Further research into a potential prognostic tool would require either a prospective analysis or a multi-institution validation cohort. A prospective study may not be feasible due to the practical hurdles of accrual (between ten and 50 times the number of patients would need to be enrolled per rare progression event) and the significant monitoring lead time to identify progressors. Resources may limit performance of such a study. A multi-institution validation cohort may be better suited to the issues at hand, namely identifying which genes in this initial study are truly predictive and which may represent overfitting.

## MATERIALS AND METHODS

### This was an institutional review board approved study

Patients with initial low-grade tumors who had a subsequent high-grade tumor recurrence (progression cohort) or recurrent low grade tumors (control cohort) were identified from a retrospective bladder cancer database from 2006 to 2016. Patients were identified as low or high grade using World Health Organization 2004 criteria. A total of 322 patients were screened with initial diagnosis of non-muscle-invasive disease and seven patients were identified as having progression from low-grade to high-grade disease, as well as six with recurrent low grade tumors only. In order to be eligible for analysis there needed to be sufficient tissue from both the low grade and high grade tumor as well as ability to obtain a blood sample to obtain normal DNA for analysis.

Five progression patients and six non-progressors were alive, had available tissue and blood, and were able to give consent and agreed to participate. Clinicopathologic data including date of diagnosis, operative management, pathologic cell type (to exclude variant histologies), pathologic stage and grade, and time for progression from initial low-grade to high-grade disease were reviewed. Time to progression was defined as the time between resection of an initial low-grade tumor to resection of a subsequent high-grade tumor. Diagnostic formalin fixed paraffin embedded tissue blocks were obtained from the Department of Pathology at the University of Texas Southwestern Medical Center, and DNA was extracted for whole exome sequencing. Buffy coat was retrieved from blood samples given for the study.

Exome sequencing was performed by the McDermott Next-Generation Sequencing Core at UT Southwestern. Sequencing libraries were prepared from 3 μg DNA with the SureSelectXT2 HSQ Reagent kit (Agilent). In brief, first DNA quality was assessed via 1.8% agarose gel and concentration were be quantified with the Qubit^®^ 2.0 Fluorometer (Invitrogen). DNA samples were sheared on the Covaris S-2 sonicator and end repaired. 3› ends of the fragments were adenylated and barcoded with precapture indexing adapters. After amplification and purification, the fragment library size distribution was checked on the Agilent 2100 BioAnalyzer, and concentration was determined by qPCR. Libraries were pooled in equimolar amounts and captured with the SureSelectXT2 Target Enrichment System. After capture and purification, samples were run on the Agilent 2100 BioAnalyzer, and final concentration was determined by qPCR. Samples were run on the Illumina HiSeq2500 using 100 nucleotide paired end SBS chemistry, with ~100-fold coverage across the targeted regions for each sample. Image intensities were processed using the HiSeq Control Software (Illumina) with default settings to generate base calls with quality metrics. Sequence reads were mapped to the reference genome (hg19) using the Burrows-Wheeler Alignment Tool (BWA). Because of the high sequence coverage across targeted regions for each sample, the alignment was processed further using SAM tools and the Genome Analysis Toolkit (Broad Institute) to confidently identify and annotate sequence variation using the RefSeq database. Somatic mutations were identified based on their presence in the tumor samples only, and annotated for their effect on the protein (missense, nonsense, frameshift, splice site) using SIFT, PolyPhen-2, and MuTect. Unsupervised clustering was performed on a correlation matrix computed from presence or absence of point mutations at the SNP level and gene level. Pearson correlation values were used to calculate a distance matrix between samples and hierarchical clustering was performed using the “Ward.D2” method and visualized in a heat map using the “gplots” function in R. Buffy coat was used for subtraction of germline mutations.

## CONCLUSIONS

Bladder cancer is a disease where focus on molecular alterations can improve future management. Tumor grade progression occurred with both high mutational homology and low mutational homology, which may represent both true tumor progression and de-novo growth, and a unique subsets of genes may be predictive for progression.

## SUPPLEMENTARY MATERIALS TABLES



## References

[1] Siegel RL, Miller KD, Jemal A (2016). Cancer statistics, 2016. CA Cancer J Clin.

[2] Castillo-Martin M, Domingo-Domenech J, Karni-Schmidt O, Matos T, Cordon-Cardo C (2010). Molecular pathways of urothelial development and bladder tumorigenesis. Urol Oncol.

[3] Sylvester RJ, van der Meijden AP, Oosterlinck W, Witjes JA, Bouffioux C, Denis L, Newling DW, Kurth K (2006). Predicting recurrence and progression in individual patients with stage Ta T1 bladder cancer using EORTC risk tables: a combined analysis of 2596 patients from seven EORTC trials. Eur Urol.

[4] Hernandez V, Alvarez M, de la Pena E, Amaruch N, Martin MD, de la Morena JM, Gomez V, Llorente C (2009). Safety of active surveillance program for recurrent nonmuscle-invasive bladder carcinoma. Urology.

[5] Ro JY, Staerkel GA, Ayala AG (1992). Cytologic and histologic features of superficial bladder cancer. Urol Clin North Am.

[6] Chang SS, Boorjian SA, Chou R, Clark PE, Daneshmand S, Konety BR, Pruthi R, Quale DZ, Ritch CR, Seigne JD, Skinner EC, Smith ND, McKiernan JM (2016). Diagnosis and Treatment of Non-Muscle Invasive Bladder Cancer: AUA/SUO Guideline. J Urol.

[7] Miyamoto H, Brimo F, Schultz L, Ye H, Miller JS, Fajardo DA, Lee TK, Epstein JI, Netto GJ (2010). Low-grade papillary urothelial carcinoma of the urinary bladder: a clinicopathologic analysis of a post-World Health Organization/International Society of Urological Pathology classification cohort from a single academic center. Arch Pathol Lab Med.

[8] Kamat AM, Karam JA, Grossman HB, Kader AK, Munsell M, Dinney CP (2011). Prospective trial to identify optimal bladder cancer surveillance protocol: reducing costs while maximizing sensitivity. BJU Int.

[9] Kawanishi H, Takahashi T, Ito M, Watanabe J, Higashi S, Kamoto T, Habuchi T, Kadowaki T, Tsujimoto G, Nishiyama H, Ogawa O (2006). High throughput comparative genomic hybridization array analysis of multifocal urothelial cancers. Cancer Sci.

[10] Kawanishi H, Takahashi T, Ito M, Matsui Y, Watanabe J, Ito N, Kamoto T, Kadowaki T, Tsujimoto G, Imoto I, Inazawa J, Nishiyama H, Ogawa O (2007). Genetic analysis of multifocal superficial urothelial cancers by array-based comparative genomic hybridisation. Br J Cancer.

[11] Letouze E, Allory Y, Bollet MA, Radvanyi F, Guyon F (2010). Analysis of the copy number profiles of several tumor samples from the same patient reveals the successive steps in tumorigenesis. Genome Biol.

[12] Bakkar AA, Wallerand H, Radvanyi F, Lahaye JB, Pissard S, Lecerf L, Kouyoumdjian JC, Abbou CC, Pairon JC, Jaurand MC, Thiery JP, Chopin DK, de Medina SG (2003). FGFR3 and TP53 gene mutations define two distinct pathways in urothelial cell carcinoma of the bladder. Cancer Res.

[13] Lamy P, Nordentoft I, Birkenkamp-Demtroder K, Thomsen MB, Villesen P, Vang S, Hedegaard J, Borre M, Jensen JB, Hoyer S, Pedersen JS, Orntoft TF, Dyrskjot L (2016). Paired Exome Analysis Reveals Clonal Evolution and Potential Therapeutic Targets in Urothelial Carcinoma. Cancer Res.

[14] Sanchez-Carbayo M, Socci ND, Lozano J, Saint F, Cordon-Cardo C (2006). Defining molecular profiles of poor outcome in patients with invasive bladder cancer using oligonucleotide microarrays. J Clin Oncol.

[15] Mitra AP, Pagliarulo V, Yang D, Waldman FM, Datar RH, Skinner DG, Groshen S, Cote RJ (2009). Generation of a concise gene panel for outcome prediction in urinary bladder cancer. J Clin Oncol.

[16] Birkhahn M, Mitra AP, Williams AJ, Lam G, Ye W, Datar RH, Balic M, Groshen S, Steven KE, Cote RJ (2010). Predicting recurrence and progression of noninvasive papillary bladder cancer at initial presentation based on quantitative gene expression profiles. Eur Urol.

[17] Bartsch G, Mitra AP, Cote RJ (2010). Expression profiling for bladder cancer: strategies to uncover prognostic factors. Expert Rev Anticancer Ther.

[18] Seiler R, Lam LL, Erho N, Takhar M, Mitra AP, Buerki C, Davicioni E, Skinner EC, Daneshmand S, Black PC (2016). Prediction of Lymph Node Metastasis in Patients with Bladder Cancer Using Whole Transcriptome Gene Expression Signatures. J Urol.

[19] Lopez de Maturana E, Picornell A, Masson-Lecomte A, Kogevinas M, Marquez M, Carrato A, Tardon A, Lloreta J, Garcia-Closas M, Silverman D, Rothman N, Chanock S, Real FX (2016). Prediction of non-muscle invasive bladder cancer outcomes assessed by innovative multimarker prognostic models. BMC Cancer.

[20] Liu Y, Noon AP, Aguiar Cabeza E, Shen J, Kuk C, Ilczynski C, Ni R, Sukhu B, Chan K, Barbosa-Morais NL, Hermanns T, Blencowe BJ, Azad A (2014). Next-generation RNA sequencing of archival formalin-fixed paraffin-embedded urothelial bladder cancer. Eur Urol.

[21] Shen J, Noon A, Liu Y, Kuk C, Ilczynski C, Ni R, Sukhu B, Chan K, Gunaratne A, Erlich A, Cremer C, Morris Q, Barbosa-Morais NL (2016). Molecular tumor grading of non muscle invasive bladder cancer based on whole tran scriptome analysis. Journal of Clinical Oncology.

[22] Prout GR, Barton BA, Griffin PP, Friedell GH, The National Bladder Cancer Group (1992). Treated history of noninvasive grade 1 transitional cell carcinoma. J Urol.

[23] Mossanen M, Gore JL (2014). The burden of bladder cancer care: direct and indirect costs. Curr Opin Urol.

[24] Lotan Y, Svatek RS, Sagalowsky AI (2006). Should we screen for bladder cancer in a high-risk population?: A cost per life-year saved analysis. Cancer.

[25] Mbeutcha A, Lucca I, Mathieu R, Lotan Y, Shariat SF (2016). Current Status of Urinary Biomarkers for Detection and Surveillance of Bladder Cancer. Urol Clin North Am.

