# Supplementary material for: Grade progression in urothelial carcinoma can occur with high or low mutational homology: a first-step toward tumor-specific care in initial low-grade bladder cancer
Source: Oncotarget. 2018 Jan 6;9(10):9415–24. doi: 10.18632/oncotarget.24072 (PMC5823658; doi:10.18632/oncotarget.24072)
Supplement: Supplementary file 1 [file oncotarget-09-9415-s001.pdf]

Grade progression in urothelial carcinoma can occur with high or low mutational homology: a first-step toward tumor-specific care in initial low-grade bladder cancer

SUPPLEMENTARY MATERIALS

Supplementary Table 1: Tumor characteristics and patient characteristics of cohort

Patient	Age at initial Diagnosis	Sex	Use of Adjuvant Intravesical Therapy (Mitomycin C)	Location Initial Low Grade Diagnosis	Location Subsequent High Grade Diagnosis (pathology)	Unifocal or Multifocal Primary	Size of Primary	Patient History of Smoking	Industrial or Other Carcinogen Exposure
**1**	34	M	Yes	Left lower bladder wall	Right superior bladder wall (HG Ta)	Multifocal	Large (> 5 cm)	None	None
**2**	74	F	No	Medial to right ureteral orifice	Right posterior bladder wall and trigone (CIS)	Multifocal	Small (< 2 cm)	Yes	None
**3**	68	F	No	Left bladder base and left lateral wall	Left ureteral orifice and left lateral wall (HG Ta)	Multifocal	Large (> 5 cm)	None	None
**4**	68	M	No	Right posterior wall	Right posterior wall, bladder base and trigone (T2)	Unifocal	Medium (2–5 cm)	Yes	None
**5**	86	M	Yes	Anterior bladder wall and left trigone	Anterior bladder wall (T1)	Multifocal	Small (< 2 cm)	Yes	None
**Control 1**	55	M	No	Left lateral wall		Unifocal	Medium (2–5 cm)	Yes	None
**Control 2**	55	F	No	Left lateral wall		Unifocal	Medium (2–5 cm)	Yes	None
**Control 3**	57	M	No	Right bladder neck		Multifocal	Medium (2–5 cm)	Yes	None
**Control 4**	56	M	Yes	Left lateral wall and base		Multifocal	Small (< 2 cm)	Yes	None
**Control 5**	65	M	Yes	Left wall		Multifocal	Large (> 5 cm)	Yes	None
**Control 6**	64	M	No	Base, posterior wall, L ureteral orifice		Multifocal	Large (> 5 cm)	No	None

Supplementary Table 2: Coverage statistics for the whole exome sequencing data (exome for genomic DNA extracted from blood is depicted in boldface)

Sample ID	Mean fold coverage	Percentage of bases covered ≥ 10 fold	Percentage of bases covered ≥ 20 fold	Percentage of bases covered ≥ 50 fold
**Patient 1**	105	99%	96%	81%
**Patient 1**	110	99%	95%	78%
**Patient 2**	99	98%	95%	78%
**Patient 2**	119	99%	97%	84%
**Patient 3**	139	99%	98%	90%
**Patient 3**	137	99%	97%	87%
**Patient 4**	142	99%	97%	84%
**Patient 4**	143	99%	95%	75%
**Patient 5**	129	99%	94%	70%
**Patient 5**	139	99%	95%	76%
**Patient 6**	111	99%	96%	84%
**Patient 7**	116	99%	97%	85%
**Patient 8**	123	99%	97%	86%
**Patient 9**	121	99%	97%	86%
**Patient 10**	130	99%	97%	87%
**Patient 11**	124	99%	97%	87%
PT 1 Blood	**120**	**99%**	**98%**	**88%**
PT 2 Blood	**147**	**98%**	**96%**	**88%**
PT 3 Blood	**130**	**99%**	**98%**	**90%**
Pt 4 Blood	**135**	**99%**	**98%**	**91%**
Pt 5 Blood	**136**	**99%**	**98%**	**91%**
Pt 6 Blood	**120**	**99%**	**98%**	**88%**
Pt 7 Blood	**133**	**99%**	**98%**	**90%**
Pt 8 Blood	**157**	**99%**	**98%**	**93%**
Pt 9 Blood	**146**	**99%**	**98%**	**92%**
Pt 10 Blood	**121**	**99%**	**98%**	**88%**
Patient 11 Blood	**94**	**99%**	**96%**	**81%**

Supplementary Table 3: Genes identified in SNVs of common origin in Patient 4

Gene	Effect	Original Nucleotide	Nucleotide Mutation
**CHSY3**	SILENT	G	C
**GPR98**	NONSENSE	C	T
**KRTAP19-8**	NONSENSE	C	T
**STAG2**	NONSENSE	C	T
**AFM**	NONE	T	A
**FAR2P3**	NONE	C	T
**FCGR2A**	NONE	C	T
**IMP4**	NONE	G	A
**MMP2**	NONE	G	A
**PPIG**	NONE	T	G
**SAP130**	NONE	C	T
**UNK (CH9)**	NONE	C	T
**HRNR**	MISSENSE	G	T
**HRNR**	MISSENSE	G	C
**LPHN1**	MISSENSE	G	T
